# Detection of Micro-Defects on Metal Screw Surfaces Based on Deep Convolutional Neural Networks

**DOI:** 10.3390/s18113709

**Published:** 2018-10-31

**Authors:** Limei Song, Xinyao Li, Yangang Yang, Xinjun Zhu, Qinghua Guo, Huaidong Yang

**Affiliations:** 1 Key Laboratory of Advanced Electrical Engineering and Energy Technology, Tianjin Polytechnic University, Tianjin 300387, China; songlimei@tjpu.edu.cn (L.S.); lixinyao211314@gmail.com (X.L.); xinjunzhu@tjpu.edu.cn (X.Z.); 2 College of Mechanical Engineering, Tianjin University of Technology and Education, Tianjin 300222, China; Yan_gangYang@163.com; 3 School of Electrical, Computer and Tele Communications Engineering, University of Wollongong, Wollongong, NSW 2500, Australia; 4 Department of Precision Instrument, Tsinghua University, Beijing 100084, China; yanghd@tsinghua.edu.cn

**Keywords:** metal screw surface, deep convolutional neural network, micro-defect detection

## Abstract

This paper proposes a deep convolutional neural network (CNN) -based technique for the detection of micro defects on metal screw surfaces. The defects we consider include surface damage, surface dirt, and stripped screws. Images of metal screws with different types of defects are collected using industrial cameras, which are then employed to train the designed deep CNN. To enable efficient detection, we first locate screw surfaces in the pictures captured by the cameras, so that the images of screw surfaces can be extracted, which are then input to the CNN-based defect detector. Experiment results show that the proposed technique can achieve a detection accuracy of 98%; the average detection time per picture is 1.2 s. Comparisons with traditional machine vision techniques, e.g., template matching-based techniques, demonstrate the superiority of the proposed deep CNN-based one.

## 1. Introduction

Due to imperfect manufacturing processes, various defects may occur in products, e.g., pinholes, scratches, cracks, etc. [1,2,3,4,5]. It is important to develop efficient automatic defect detection techniques for quality control of products, or for pinpointing faulty parts. This work focuses on surface defect detection of metal screws. At present, most screw manufacturers carry out quality control through sampling, and the screw samples are measured with a caliper or an optical amplifier. For screws with full inspection requirements, a lot of manpower has to be used for manual inspection. It is time-consuming and labor-intensive to manually inspect small-sized screws that are produced in large quantities. Recently, in the field of surface defect detection, various detection techniques based on image processing have been developed. Zhang [6] designed a product defect recognition system based on machine vision, where an image acquisition module is used to obtain an image of the product, which is processed to judge the degree of defect. This method requires high-quality images, and it is difficult to detect micro defects. An image recognition system to detect screw internal thread was developed by Chen [7] based on adaptive threshold segmentation and morphological opening calculation to achieve screw identification. However, strong image interference may cause serious under-segmentation, leading to poor detection accuracy. Yan [8] developed screw thread detection system-based on a CCD (Charge-Coupled Device) digital image correction technique, where maximum variance segmentation and relative sample standard were used to achieve screw identification. However, large image interference can lead to serious over-segmentation, resulting in failure of the inspection. Li et al. [9] designed a vision inspection system to capture railway road images and extract defects from projected contours. Feng et al. [10] developed an automatic defect detection method using probabilistic topic models. Marino et al. [11] used a multilayer perceptual neural classifier to detect missing hex bolts. Aytekin used a high-speed laser range finder, pixel information, and histogram similarity analysis to achieve real-time railway fastener detection [12]. Prasanna et al. [13] classified crack images by extracting the curve in the image and using SVM (support vector machine) with handcrafted feature descriptors. Later, Prasanna et al. [14] combined AdaBoost with random forest method to improve the classifier of Aytekin. In [15], based on the selection of the feature vector of defect images, BP neural networks and SVM were used for pattern recognition, but this method is not robust in extracting feature vectors, and the recognition accuracy is not high. Marco Leo et al. [16] proposed a system for automatic monitoring of welding process in dry stainless steel kegs for food storage. Cropped regions are processed by three different algorithmic procedures that perform the monitoring of welding dimensions (spatial metrology), radiometric appearance (radiometric metrology), and local shape analysis, in order to detect thin/thick penetrations, darker areas, and outgrowths respectively.

Traditional image recognition methods require high illumination conditions and have poor adaptability. It is difficult to meet the recognition requirements with surface defect images collected under different conditions. Furthermore, the traditional image detection methods mainly acquire image features through intensive image preprocessing. However, screw surface defects can be very small, resulting in features that are not clearly visible. It is difficult for traditional methods to accurately identify screw surface defects.

In this work, a method for identifying micro defects of metal screw surfaces is developed based on deep convolutional neural networks, and an optical platform for acquiring screw images is built. Images of defected and defect-free screw surfaces are collected, which are used to train the designed deep convolutional neural network (CNN). To enable efficient detection, we first locate screw surfaces in the pictures captured by the cameras, so that the images of screw surfaces can be extracted, which are then input to the CNN-based defect detector. The proposed method does not need to acquire the features of the screw surface images in advance, and is robust to illumination changes. Comparisons with traditional machine vision techniques, e.g., template matching-based techniques, demonstrate the superiority of the proposed deep CNN-based one.

## 2. Method

### 2.1. System Description

**Figure 1:**
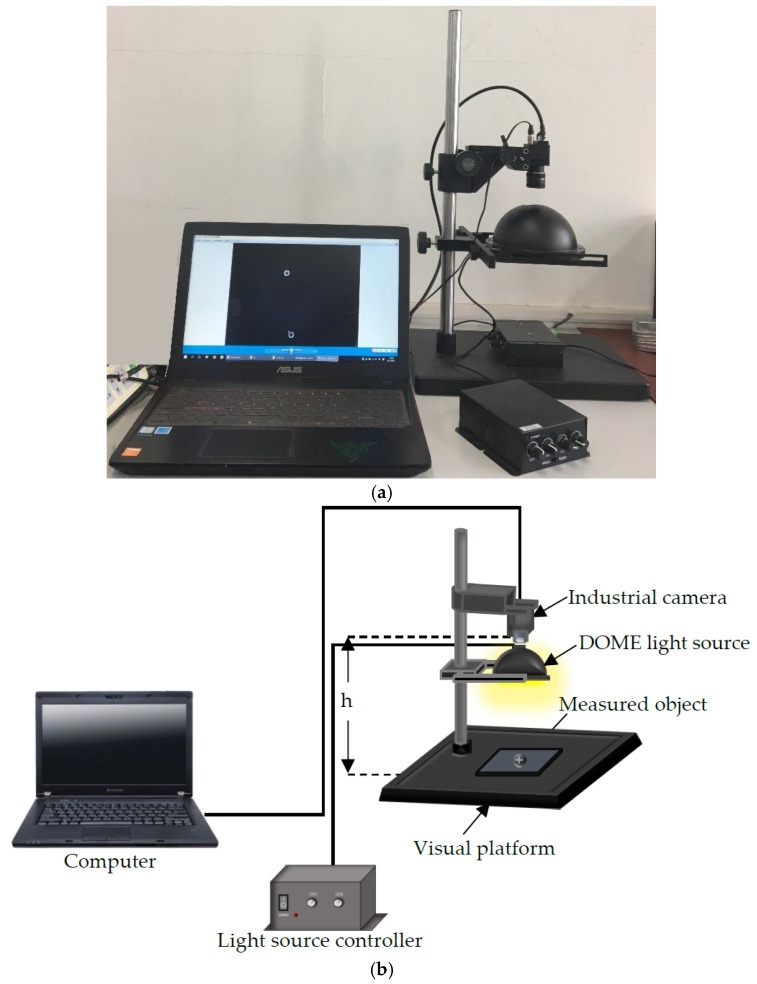
Experimental system for image acquisition. (**a**) Detection system, (**b**) System structure diagram.

Figure 1 shows the optical experimental platform system for acquiring screw images. We use an industrial camera RS-A2300-GC50 (manufactured by China Microview, Beijing, China) with a CMOS resolution of 1600 × 1200 pixels and a 16 mm (M0814-MP2) lens. The distance between the camera and the object h is about 200 mm. The light source controller controls the brightness of the DOME light source. The large opening angle of the DOME light source is helpful for uneven surface imaging, and multiple reflections through the inner wall of the hemisphere can completely eliminate shadows, which is helpful for metal or mirror surface inspection. The captured image is a 24-bit image of size 1600 × 1200 pixels in BMP format. The length of a pixel in the image is approximately 0.0765 mm (i.e., 122.35 mm/1600 pixels = 0.0765 mm/pixel).

### 2.2. Data Preparation

**Figure 2:**
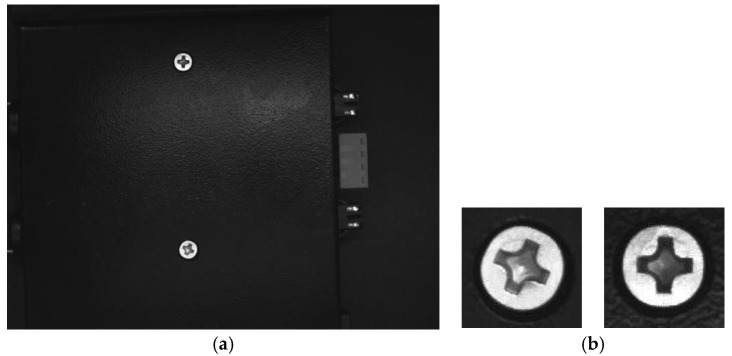
Data acquisition. (**a**) Original image, (**b**) Detected screws.

**Figure 3:**
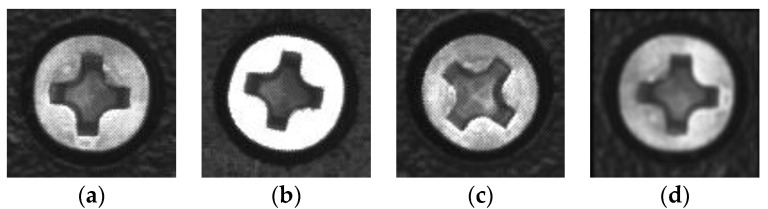
Data enhancement. (**a**) Original image, (**b**) image obtained by adjusting the light intensity, (**c**) rotated image, (**d**) distorted image.

**Figure 4:**
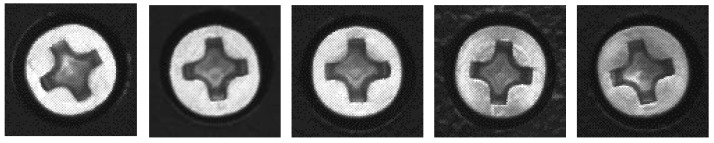
Defect-free screws.

**Figure 5:**
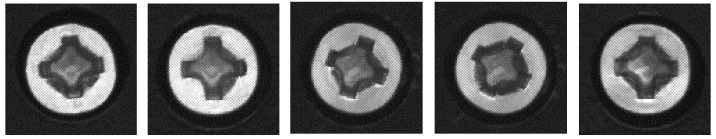
Stripped screws.

**Figure 6:**
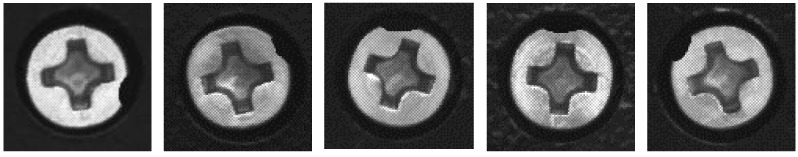
Surface-damaged screws.

**Figure 7:**
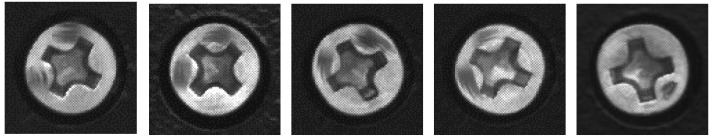
Surface dirty screws.

The metal screws used in this study are GB819 cross countersunk head screw M3s with a diameter of 5.2 mm. The types of surface defects considered in this work include dirt on surface, surface damage, and stripped screws. An example of the image captured by the detection system is shown in Figure 2a. The contours of the screws are obtained through image contour query, and the region of interest is selected to obtain the screw images shown in Figure 2b where the sample size is 96 × 96, which is captured under different illuminations. The training sample set consists of 230 defect-free screws, 287 stripped screws, 205 surface-damaged screws, and 256 surface dirty screws. A large number of extended samples are generated through translation and distortion. The expanded sample set has a total of 3000 samples. Different lighting conditions are achieved by adjusting the light source controller. Figure 3 shows the effect of adjusting the light intensity, rotation, and distortion of the same screw image. Some randomly-selected defect-free samples are shown in Figure 4, and some samples of defective screws are shown in Figure 5, Figure 6 and Figure 7.

### 2.3. Neural Network Structure

In this work, we propose the use of convolutional neural networks (CNN) to design the detection method. The essence of CNN is to use the huge data to filter the image multiple times through and reduce the dimension by downsampling. In addition, the nonlinear fitting of the activation function is used to obtain more abstract and deeper essential features of the target, so as to realize the recognition of the target and solve the important problem of manual design features in the past. Therefore, a deep learning model based on convolutional neural network is suitable for image processing and other related machine learning problems.

**Figure 8:**
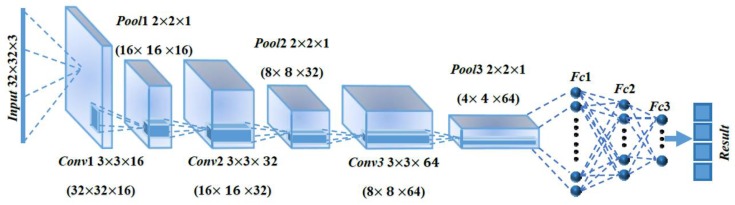
Architecture of the CNN used in this paper.

The metal screw surface defect detection method in this paper is developed based on the traditional LeNet-5 [17]. The architecture of the network is shown in Figure 8. The first layer is the input layer with size 32 × 32 × 3 (i.e., the length and width of the images are 32, and the number of color channels is 3). The input data pass through the architecture and are generalized with spatial size reduction to 4 × 4 × 64 at Pool 3; the output of the layer is then fed into the rectified linear unit (ReLU) layer. Finally, the softmax layer outputs the probabilities of the four cases: defect-free screw, dirty screw, damaged screw, and stripped screw. A dropout layer is located after each layer of Fc1, Fc2, and Fc3. In order to keep the dimension unchanged after convolution, zero padding is used to get better results in the final feature search without affecting the operation speed.

### 2.4. Detection Method

**Figure 9:**
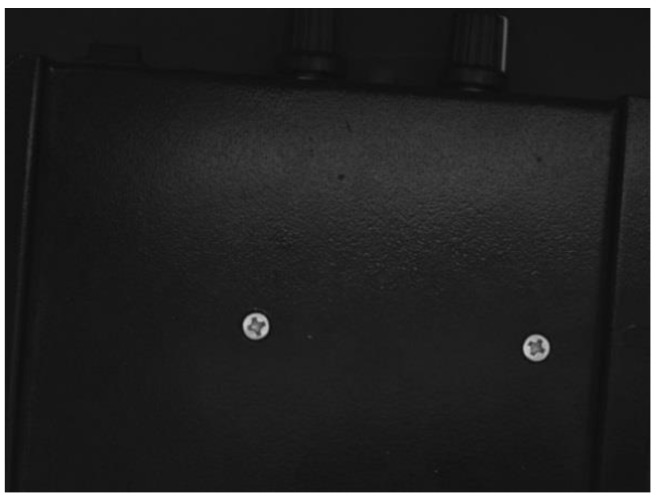
Original image captured by the camera.

**Figure 10:**
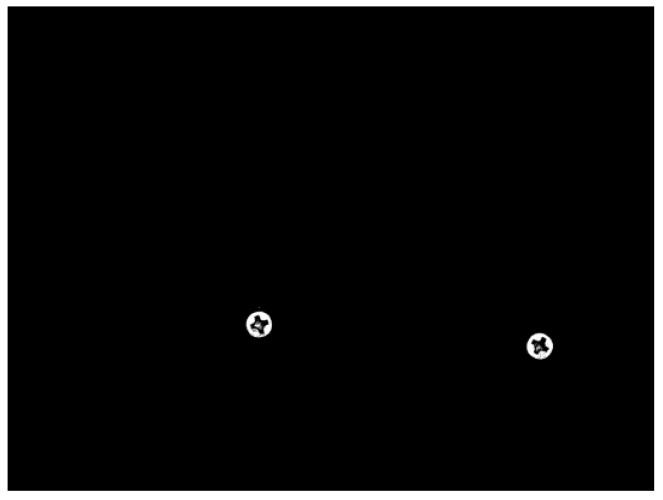
Binarized image.

**Figure 11:**
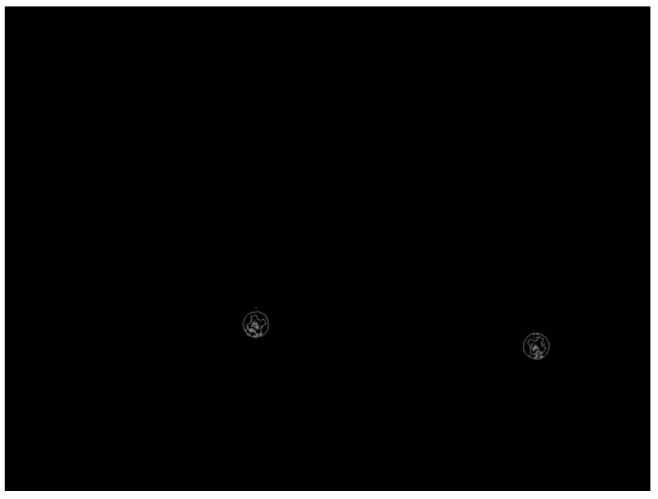
Contours query.

**Figure 12:**
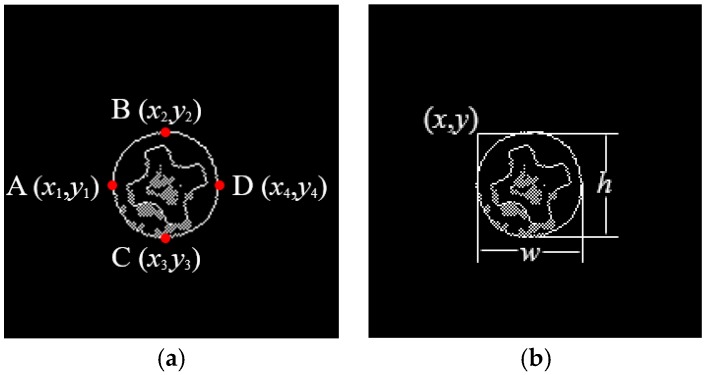
Screw surface localization. (**a**) Boundary points, (**b**) Located screw surface area.

**Figure 13:**
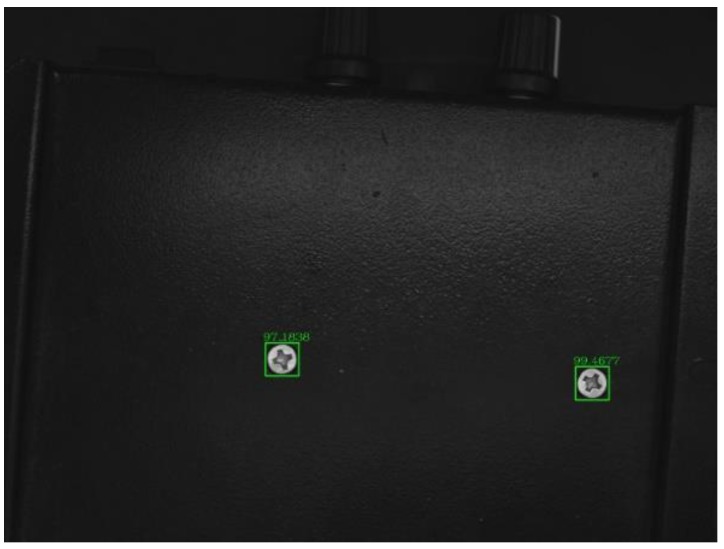
Detection results.

To efficiently detect the defects on screw surfaces, the first step is to locate the screw surfaces in the images (as shown in Figure 9) captured by the industrial camera. Then, the screw surface images are extracted, which are fed to the trained deep CNN for defect detection. The screw surface defect detection method is summarized as follows:

1. Use the optical platform to collect the object image, which may contain multiple screw surfaces, as shown in Figure 9.
2. Carry out gray-scale processing, which turns the three-channel color images into single-channel gray-level image;
3. The gray image of screw is binarized, i.e., 0–255 gray image is converted into 0 (black) or 255 (white) image, as shown in Figure 10;
4. Through image contour query [18], get the contours of the screw surfaces, as shown in Figure 11;
5. Obtain the positions, heights and widths of the screw surfaces in the image based on their contours. Take the screw in Figure 12 as an example. In Figure 12a, points A, B, C, and D are the leftmost, uppermost, lowermost, and rightmost points of the screw respectively. Then, the position (*x*, *y*), the height *h*, and the width *w* of the screw are obtained, i.e., *x* = $x_{1}$, *y* = $y_{1}$, *h* = |$y_{2}$ − $y_{3}$|, *w* = |$x_{4}$ − $x_{1}$| as shown in Figure 12b;
6. Based on the position, height, and width of each screw surface, extract the color images of the screw surface from the original image captured in Step 1. The size of the image is adjusted to 32 × 32, and the image is then input to the trained CNN for defect detection;
7. The screw positions obtained in Step 5 are marked on the original image, and the defect types are also indicated with different color borders. Figure 13 shows an example.

## 3. Experiments

Determining appropriate hyperparameters (e.g., learning rate and regularization parameters) is cumbersome, and there is no accurate guidance for optimizing those parameters. Therefore, these parameters are obtained through trial and error, guided by checking the verification set error [19]. In order to demonstrate the superiority of the CNN proposed in this paper, it is compared with the traditional LeNet-5. The computer used in the experiment is an ASUS notebook ROG GX501VIK7700 with the configuration shown in Table 1. The CNN trained the model with tensorflow and Google’s machine learning architecture.

**Figure 14:**
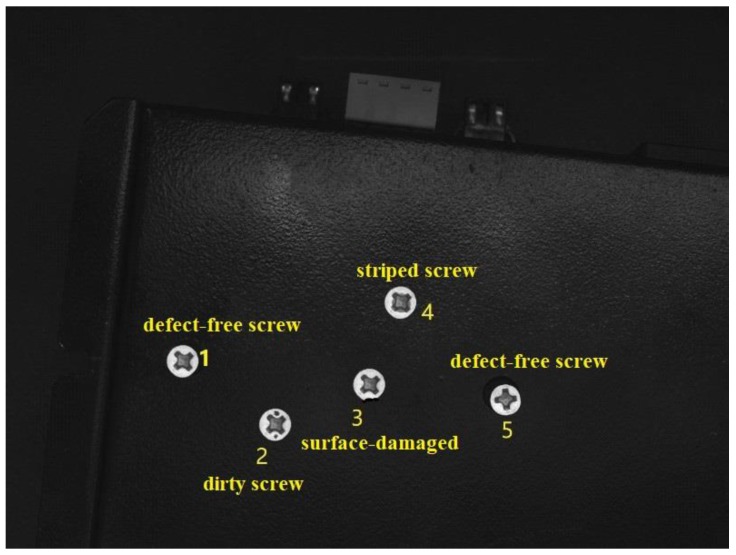
Screws for testing.

**Figure 15:**
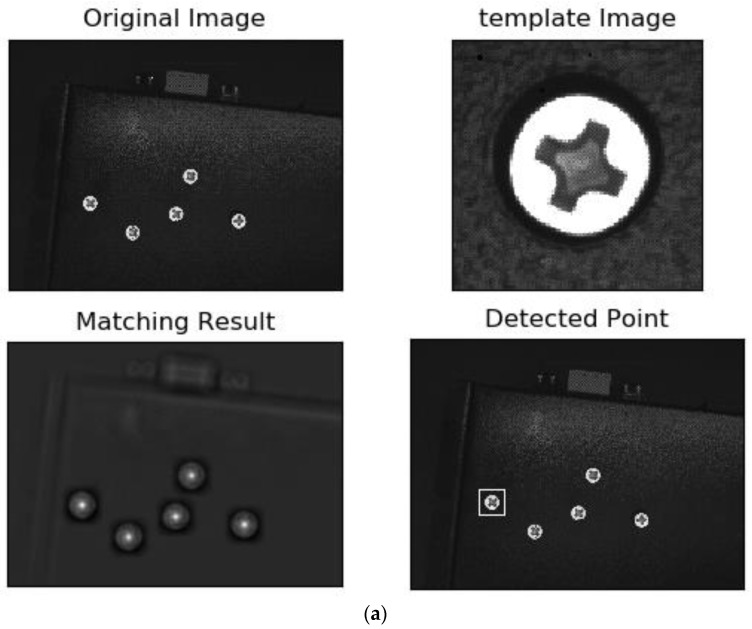

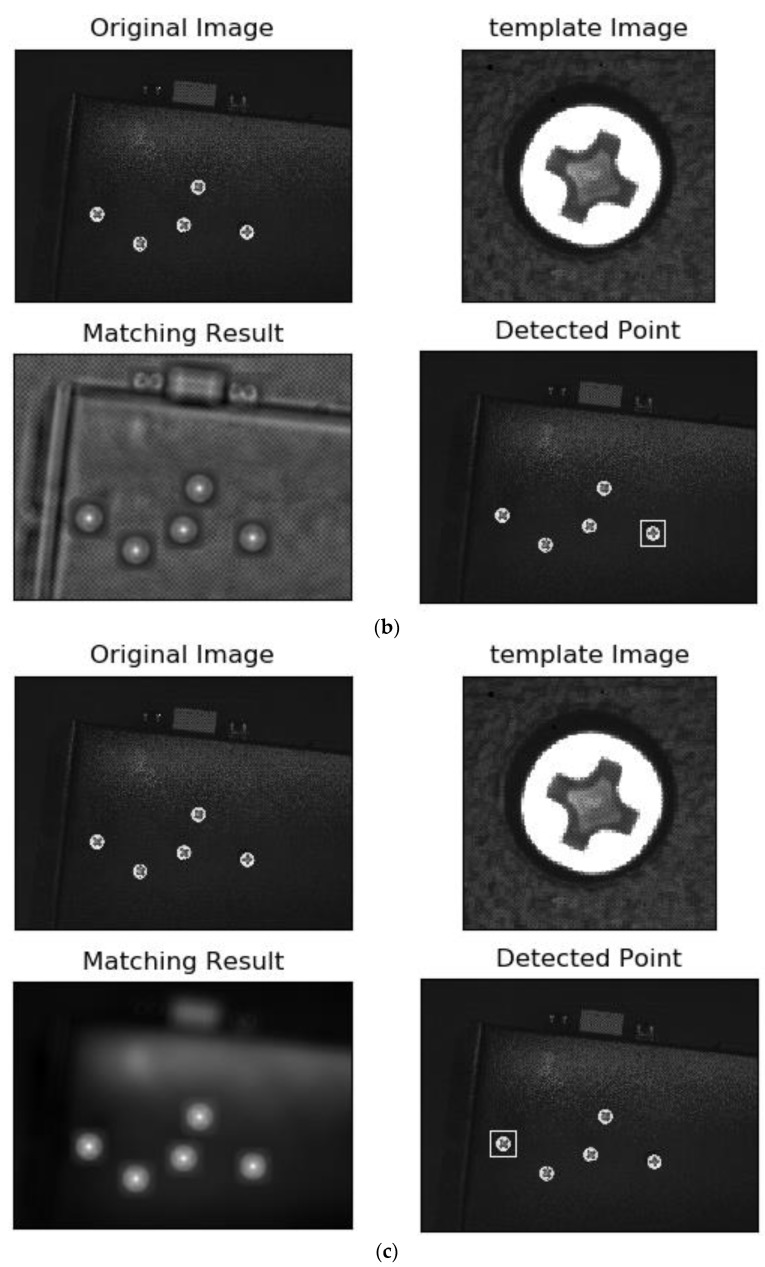

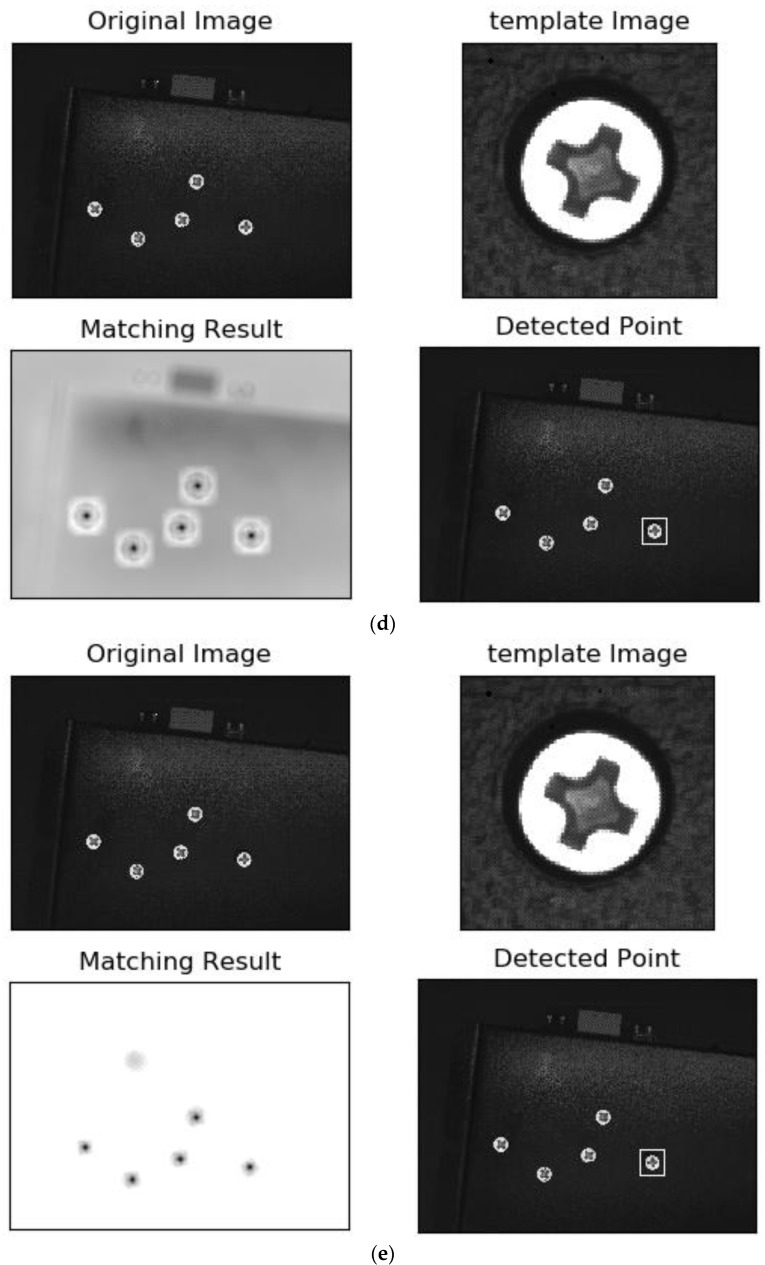
Traditional template matching-based detection methods. (**a**) Normalized correlation matching method; (**b**) Normalized correlation coefficient matching method; (**c**) Correlation coefficient matching method; (**d**) Squared difference matching method; (**e**) Correlation matching method.

Firstly, we used the traditional method to detect the screw defects. Figure 14 is the original image of the object to be detected (the yellow numbers are post-marked to ease the description of the screw surfaces.). There are five screws in the image: Screws 1 and 5 are defect-free screws, 2 is a dirty screw, 3 is a surface-damaged screw, and 4 is a striped screw. We selected an image of a defect-free screw as a template, and used various template-matching methods for defect detection, including the normalized correlation matching method [20], normalized correlation coefficient matching method [21], correlation coefficient matching method [22], normalized square difference matching method [23], square difference matching method [24], and correlation matching method [25]. The results are shown in Figure 15. It can be seen that the template matching method cannot accurately detect the two defect-free screws.

**Figure 16:**
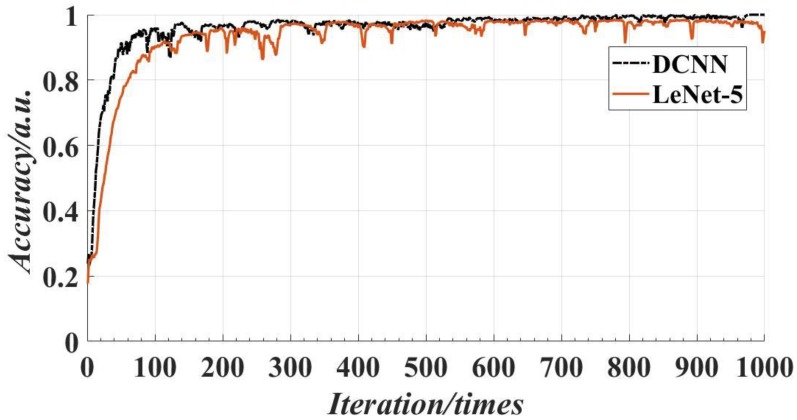
The accuracy of the two models with 1000 iterations.

**Figure 17:**
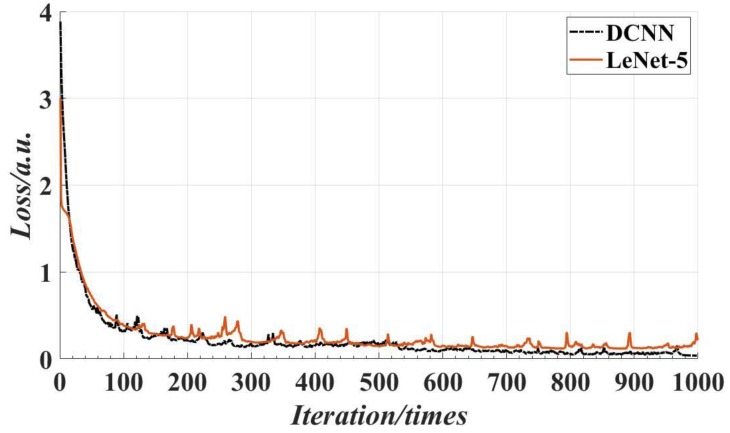
The minimum training loss of the two models with 1000 iterations.

**Figure 18:**
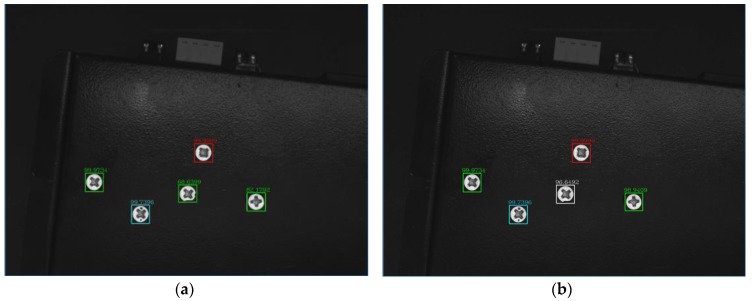
The test results of two models. (**a**) The test results of LeNet-5; (**b**) The test results of the proposed DCNN.

In order to further verify the superiority of the proposed model, the proposed CNN was compared with the traditional LeNet-5. The same screw data set is used to train the two networks. Experiments showed that the accuracy and loss tend to be stable after 1000 iterations. Figure 16 shows the accuracy of the traditional LeNet-5 and the proposed deep CNN with 1000 iterations. It can be seen that the accuracy of the proposed DCNN is much higher than the traditional LeNet-5 at the beginning of the training, and the accuracy of the training was close to 100% with 550 iterations, and about 100% accuracy was achieved with 800 iterations. Figure 17 shows the minimum training loss of the traditional LeNet-5 and the proposed DCNN with 1000 iterations. It can be seen from the figure that the loss rate of the proposed DCNN decreases rapidly, which is slightly better than that of the LeNet-5. When the number of iterations is about 550, the loss rate is close to 0. Figure 18 shows the detection results of the traditional LeNet-5 and the proposed DCNN, where the traditional LeNet-5 has a detection error.

To better examine the detect performance of the two networks, the same verification set is used to test the accuracy of the two methods, and 1000 different types of images are selected as the test set. The results are shown in Table 2.

A comparison of different CNNs is shown in Table 3.

## 4. Conclusions

In this work, we have developed a deep CNN-based method to detect micro defects of metal screw surfaces. Experimental results demonstrated the superiority of the proposed method, compared to the traditional template matching methods and the LeNet-5-based method. It has been shown that the proposed method can achieve a detection accuracy of 98%. The proposed method may also be used in other industrial production inspection applications, such as bottle cap defect detection, mobile phone screen defect detection, etc.

## Figures and Tables

**Table 1:** Computer configuration.

CPU Model	Intel Core i7 7700HQ
Core/thread number	Four core/eight threads
Memory capacity	16 GB
Hard drive capacity	1 TB
Graphics chip	NVIDIA GeForce GTX 1080 Max-Q
Video memory	8 GB

**Table 2:** Recognition rate of different categories of samples in the test set.

	Total Number of Images	Correct Detection	Error Detection	Accuracy
LeNet-5	1000	958	42	95.8%
The proposed DCNN	1000	984	16	98.4%

**Table 3:** The comparison of different CNN.

	Time	Accuracy
YOLO	Faster	Low
R-CNN	Low	Low
Faster-RCNN	Fast	High
SSD	Low	Higher
The proposed DCNN	Faster	Higher
